# In this month’s Bulletin

**DOI:** 10.2471/BLT.16.000516

**Published:** 2016-05-02

This month’s issue has a special theme which focuses on the implementation of the *Global strategy for women’s, children’s and adolescents’ health (2016–2030). *

In the editorial section, Flavia Bustreo et al. (310) highlight the ongoing need to analyse what works to achieve the objectives of this strategy: survive, thrive and transform. Ian Askew et al. (311) remind readers why sexual and reproductive health and rights need to be addressed in the response to health emergencies. Zanele Mabaso et al. (312) ascribe the inclusion of adolescent health outcomes in the global strategy to young people’s participation, and Nila Moeloek & Kesetebirhan Admasu (313) explain how ministers of health can help ensure positive change in their countries. 

Bethlehem Kiros (316–317) reports on efforts to improve health outcomes for women and children living in rural Ethiopia. Sophie Cousins (318–319) describes how chances of survival during pregnancy and childbirth have greatly improved in Nepal. Joannie Bewa tells Fiona Fleck (320–321) how campaigns for sex education and free contraception are changing reproductive health prospects for young people in Benin.

## Bangladesh, Cambodia, China, Egypt, Ethiopia, the Lao People’s Democratic Republic, Nepal, Peru, Rwanda and Viet Nam

### Achieving women’s and children’s health goals

Syed Masud Ahmed et al. (351–361) analyse national success factors.

## India

### Improved reporting of infant deaths, maternal deaths and stillbirths

Preeti H Negandhi et al. (370–375) describe a maternal infant death review system.

### Getting help when it’s most needed

Jennifer A Newberry et al. (388–392) introduce an emergency response line for women confronting violence.

## Liberia

### Where there are few doctors

Obed Dolo et al. (383–387) describe a programme to train midwives to do surgical procedures.

## Mexico 

### Analysing maternal health services and outcomes

Teresa Murguía-Peniche et al. (322–330) study trends and distributions of stillbirths.

Margaret C Hogan et al. (362–369) reclassify causes of maternal deaths.

## Global

### Uncovering hidden determinants of health

Jeanne Chai et al. (331–339) study the association between child growth and violence in the home.

### Adolescent suicidal behaviours 

Britt McKinnon et al. (340–350) estimate the prevalence of suicidal ideation in 32 low- and middle-income countries.

### Transforming society for better health outcomes

Cicely Marston et al. (376–382) explain why community participation is crucial.

### Towards fair assessments

Laura Frost et al. (393–395) explain why many voices are needed to understand health and development issues. 

### Meeting strategic goals

Shyama Kuruvilla et al. (398–400) outline a roadmap for implementation.

### Eliminating violence

Claudia García-Moreno & Avni Amin (396–397) argue that development goals will only be met if violence elimination targets are reached.

### Aligning approaches to maternal and child health

Pascal Bijleveld et al. (401–404) list the challenges faced by countries.

